# Utilization of Pomelo (*Citrus maxima*) Peel Waste into Bioactive Essential Oils: Chemical Composition and Insecticidal Properties

**DOI:** 10.3390/insects13050480

**Published:** 2022-05-20

**Authors:** Naduvilthara U. Visakh, Berin Pathrose, Arunaksharan Narayanankutty, Ahmed Alfarhan, Varsha Ramesh

**Affiliations:** 1 Department of Agricultural Entomology, College of Agriculture, Kerala Agricultural University, Thrissur 680656, India; visakhnu17palazhi@gmail.com; 2 Division of Cell and Molecular Biology, PG & Research Department of Zoology, St. Joseph’s College (Autonomous), Devagiri, Calicut 673008, India; 3 Department of Botany and Microbiology, College of Science, King Saud University, P.O. Box 2455, Riyadh 11451, Saudi Arabia; alfarhan@ksu.edu.sa; 4 Department of Biotechnology, Deakin University, Burwood, VIC 3125, Australia; vramesh@deakin.edu.au

**Keywords:** essential oil, larvicidal toxicity, insecticidal toxicity, fumigant toxicity, repellent activity, limonene, *Tribolium castaneum*, *Callosobruchus maculatus*, *Armigeres subalbatus*, *Culex tritaeniorhynchus*, *Aedes aegypti*

## Abstract

**Simple Summary:**

The disposal of agricultural waste products is an emerging concern and an alternative to this is the development of value-added products from these wastes. Here we extracted the essential oil from *Citrus maxima* (CMEO) and examined its larvicidal and pest control potentials. Results pointed out that CMEO can be effective biopesticides against two major insect pests of stored grains. Furthermore, CMEO had a significant larvicidal action against different mosquito species. This study provided useful information on the compositional aspects and insecticidal properties of CMEO.

**Abstract:**

The wastes generated during the post-harvest handling of various agricultural commodities is rather under-utlilized. The peels of citrus fruits are often discarded as waste. Citrus peels are rich in essential oils and exhibit toxicity towards various insect species. The essential oils are also an eco-friendly option for insect pest management. The *Citrus maxima* peel essential oil (CMEO), a waste product, characterized it, and evaluated its potential for insect pest management. The major terpenoids present in CMEO are Limonene and α-Pinene. The CMEO displayed potentials in controlling the insect pests via contact and fumigant toxicity. Moreover, CMEO showed significant larvicidal activities against *Culex tritaeniorhynchus* and *Aedes aegypti* species of mosquitoes; however, *Armigeres subalbatus* was more resistant. The biological safety of the essential oil was also tested against the stored seeds, where no significant inhibition of seed germination was noticed compared to the control. Utilizing a waste product such as citrus peel for pest management can achieve the dual objective of waste utilization and eco-friendly pest management. Overall, the CMEO is therefore found to be a bioactive essential oil extracted from the wastes of pomelo (*C. maxima*).

## 1. Introduction

The pollution and environmental issues created by agriculture and food wastes are an important and emerging concern [1]. However, the production of bioactive by-products and secondary metabolites is an emerging area of agri-food waste management. Among these, the essential oils isolated from food wastes can have significant economic value.

Essential oils are important plant-derived compounds with potent biological activities that can be utilizable in agriculture, medicine, and biotechnology. Essential oils are also known to control the population kinetics of microbes, including bacteria [2], various viruses [3], infectious fungi [4] and so on. It is also evident that these essential oils inhibit the biofilm formation effects of various bacterial colonies [5,6]. Essential oils can also alleviate antibiotic resistance in multiple pathogenic microorganisms [7].

Besides antimicrobial properties, essential oils are also known for their potential effects on economically destructive pest species. The essential oils are emerging tools in the control of pest populations in warehoused grains. Various essential oils are effective against insect pests such as *Sitophilus* sp., *Callosobruchus* sp., *Tribolium* sp., etc. via their multi-organ toxic insults [8,9]. Apart from these, the essential oils are also known for their anticancer properties [10], antidiabetic effects [11], hypolipidemic effects [12], and several other pharmacological effects. Citrus peels are one of the major agriculture waste as well as an worthy source of essential oils that can be commercially exploited as an eco-friendly pest management [13]. Though there are several beneficial effects for the fruits of various citrus plants, their peels are always emerging as the predominant waste products.

Citrus plants are well-known for its nutritional and pharmacological effects [14,15]; further these fruits are important sources of essential oils with wide application [16]. The essential oils of citrus fruits are currently being used as botanical pesticides [13]. The terpenoids present in citrus essential oils exhibit insecticidal [17], fumigant [18], and repellent [19] activity against various insects pests present in warehouse grains. Essential oils derived from citrus fruits also possess significant activity against mosquitoes [20,21,22]. Among the various species of citrus fruits, the largest one and the least studied is *Citrus maxima* (L.) Osbeck (synonym *Citrus grandis*). Studies have indicated the possible uses of *C. maxima* peels. The extracts of *C. maxima* peels exhibit radical inhibitiory and hypoglycemic properties [23,24]. Besides, the extracts of the plant peels also inhibit neuro-inflammation and subsequent cognitive impairment [25,26]. Furthermore, significant radical reducing and antimicrobial benefits of the CMEO is reported [16,27]. Further, the CMEO has been effective in the suppression of hyphal growth in *Aspergillus flavus* [28].

However, studies on the utilization of CMEO extracted from its peel (an agro-food waste) for its insecticidal and pharmacological applications are lacking when compared to other species of citrus. Therefore, here we extracted the peel essential oil of *C. maxima* (CMEO) and characterized its chemical configuration through GC-MS to identify the bioactive components. The efficacy of CMEO against the insect pests of warehoused grains and mosquitoe larvae were analyzed. The biosafety aspects of the CMEO were determined using seed germination and non-targetted organism models.

## 2. Materials and Methods

### 2.1. Collection of Citrus Peel and Essential Oil Extraction

Pomelo (*Citrus maxima*) fruits were collected from the fruit orchard at Kerala Agricultural University, Thrissur, India (10.5449° N, 76.2864° E) during December 2021. After washing these pomelo fruits with distilled water, peels were removed by using knife. Peels were then kept in sterile ziplock bags at 4 °C until they were used for essential oil extraction. Hydro-distillation of fresh peels (300 g) was carried out using modified Clevenger method for the extraction of essential oil [29]. The hydro-distillation process lasted for 5 h (100 °C). The CMEO obtained was passed through sodium sulphate (AR) to remove the content of water. On a fresh weight basis, the oil yield was determined by using the following formula yield (%, *v*/*w*):

Yield (%, *v*/*w*) = V_EO_/W_F_ × 100

where V_EO_ is the dry essential oil volume, and W_F_ is the weight of pomelo peels used for essential oil extraction. The CMEO thus obtained was put in storage using dark amber-coloured glass bottle at 4 °C inside the refrigerator until required for experiments [30].

### 2.2. Test Insects and Larval Culture

Red flour beetles (*Tribolium castaneum*) were reared using wheat flour. In order to eradicate prior insect infestation, wheat grains were heated for six hours at 50 °C and then it was milled into wheat flour. Twenty adults of *T. castaneum* were released into plastic containers (18 cm × 10 cm) containing 250 g of sterilized wheat flour fortified with 5% (*w*/*w*) brewer’s yeast. Ten such containers were prepared and kept in the culture room. After five days of oviposition, the adults were then sieved out and transferred to fresh rearing containers. Rearing containers were regularly examined to obtain uniformly aged adults (temperature 30 ± 2.5 °C; relative humidity of 80 ± 4.2%). All bioassays were conducted on adult insects (17 ± 2 days old) by sieving from wheat flour.

The culture of pulse beetle (*Callosobruchus maculatus*) was maintained on green gram grains procured from a local market. The grains were de-infestated by washing and drying 60 °C for one hour. Twenty adult male and female beetles were released into plastic containers (1 L volume) containing 100 g of sterilized green gram grains. Containers were covered with muslin cloth and kept for incubation for five days. After five days, adult insects were removed, and culture maintained (temperature 30 ± 2.5 °C; relative humidity of 80 ± 4.2%). The adult insects emerged from this culture was used for sub-culturing. For bioassays, 5 ± 2-day-old adult insects were used. All the experiments were conducted at the Pesticide Residue Testing Laboratory, Department of Agricultural Entomology, Kerala Agricultural University, Kerala, India.

The different mosquito species (*Ar. subalbatus*, *Ae. aegypti* and *Cx. tritaeniorhynchus*) were reared in cages (450 mm × 300 mm × 300 mm) under standard conditions (28 ± 2.5 °C; 75% humidity; 16 light: 8 h dark cycle). The larvae were reared in plastic trays and those in the third instar stage were collected for study.

### 2.3. Analaysis of Chemical Configuration Using GC-MS

The instrument used was TSQ 8000 Evo (Thermo scientific, Waltham, MA, USA) instrument equipped with an autosampler and TG-1MS capillary column (30 mm × 0.25 mm × 0.25 μm). The T_OV_ (temperature of oven) was sustained at 50 °C for 1 min and a temperature ramping of at 10 °C min^−1^ to 120 °C, then to 270 °C for 5 min at 5 °C min^−1^. The temperature of sample injector was retained at 250 °C. With a split ratio of 1:200, the samples (0.1 µL) were injected. Helium served as the carrier gas, with a flow rate of 1.0 mL min^−1^. The GCMS spectra (35 to 500 *m*/*z)* with a dwell time of 0.2 ms. Xcalibur 1.1 software was recorded and analyse the mass spectra data (Thermo scientific). Spectral comparison was done with NIST library (library similarity and reverse similarity index > 800), the essential oil components were identified. Calculations were made for individual chemical component peak areas to determine relative percentage amounts.

### 2.4. Contact Toxicity

The adulticidal assay was conducted by using the residual film method with few modifications [31]. Initially, the concentrations of essential oils required to obtain insect mortality were assessed over a broad range of doses. Further, a narrow range of concentrations (40–120 mg/cm^2^ for *T. castaneum* and 2–10 mg/cm^2^ for *C. maculatus*) were bioassayed to obtain LC_50_ and LC_90_. The CMEO was dissolved in acetone (HPLC grade) to obtain the desired concentration and applied to 90 mm (mg/cm^2^). A uniform film of essential oils was formed over the Petri dish by gently rotating the Petri plate. For solvent evaporation, the treated Petri dishes were air-dried for 15 min, followed by the release of 10 adult insects and then incubating the plates at 28 ± 2 °C and 80 ± 5%. Petri dishes were enclosed with perforated plastic lids to avoid any fumigant toxicity. Acetone (1 mL) was applied inside the Petri dish for control experiments. Three replications were employed for both treatments as well as the control. The percent mortality for *C. maculatus* and *T. castaneum* was measured at the end of 24 and 48 h and Mortality data was corrected using Abbott’s formula [32].

### 2.5. Fumigant Toxicity

A modified method was used to determine the fumigant toxicity of essential oils derived from *C. maxima* against *T. castaneum* and *C. maculatus* adults [33]. Initially, preliminary bioassays were done with a wide range of concentrations to fix the desired concentrations for the experiment. In brief, fumigant toxicity was accomplished by placing filter paper discs (2.5 cm diameter) impregnated with the CMEO (1, 3, 5, 7, 10 mg/L air for both *T. castaneum* and *C. maculatus*) of plant essential oils (mg/L air) hanging down by a thread in the polyacrylamide plastic containers (80 mL) containing 10 adults of *T. castaneum* and *C. maculatus*, and sealing them tightly. Another airtight polyacrylamide plastic container (80 mL) was used for the control without essential oils on the filter paper. For both treatments and controls, three replicates were employed. Mortality was recorded after 24 and 48 h from the start of essential oil exposure. The treated sets were kept at a constant temperature of 28 ± 2 °C and relative humidity of 80 ± 5%. Abbott’s equation was used to correct mortality data as mentioned earlier.

### 2.6. Repellent Activity Assay

The method of area preference was utilized to estimate the repellent activity [34]. We evaluated CMEO’s repellent activity against adult insects of *T. castaneum* and *C. maculatus*. Briefly, the test area was a 9 cm filter paper disc cut in to half. Varying concentrations of CMEO (0.5, 1.5, 2.5, 3.5 and 5 mg/cm^2^) were diluted in 0.5 mL acetone (HPLC grade) to prepare various solutions. The Petri plate was enclosed with a perforated plastic lid, and on each filter paper disc, ten adult insects were introduced to each concentration of CMEO. In each time interval (2, 4, 6, 12 and 24 h) the insects present on each half of disc was numerated. Percent repellence was calculated by aforementioned method [34].

Categorization of repellent effects by essential oils was discussed and estimated [35]. On the basis of percentage repellence (PR), five classes could be sorted: Class 0 = PR of 0 to 0.1%; Class I (0.2–10%); Class II (20.1–40%); Class III (40.1–60%); Class IV (60.1–80%), and Class V (80.1–100%).

### 2.7. Effect of CMEO on Wheat Germination

The impact of CMEO on the sprouting of wheat grains (stored products) is carried out to analyze the phytotoxic effects; briefly, the grains (25 nos) were kept in a moisturized container with access to light and water. The grains were exposed to varying concentrations (50, 100, and 250 µg/mL) of CMEO (in 0.01% Tween 80). The relative humidity of the test samples were set at 75% and the incubator was maintained at 27 ± 2.5 °C in the dark (no photoperiod). Development of a radicle of length 1 cm was considered as a true germination and noted. A control involving 0.01% Tween 80 was used to dissolve the CMEO, and therefore a vehicle control was maintained to normalize the data. All assays were repeated six times, with each conducted in triplicate.

### 2.8. Screening of Mosquito Larvicidal Activity by CMEO

The larvae of *Cx. tritaeniorhynchus*, *Ar. Subalbatus* and *Ae. aegypti* were collected in the third instar stage, and approximately 50 of them were taken to individual chambers. The CMEO (dissolved in Tween 80 at 0.01%) was added to each of the beakers (0–200 µg/mL) and observed for 24 h. Tween 80 (0.01%) was the control and the values were normalized using this group. The larvicidal assays were carried out four different times, each conducted in triplicate. The average mortality was plotted against concentration, and LC_50_ value was determined by probit analysis.

### 2.9. Toxicity on Non-Targeted Organism (Poecilia reticulata)

The toxicity of pest and vector control agents can also harm non-targeted organisms including fishes. Hence, we selected *Poecilia reticulata* as a model organism for non-targeted species toxicity. Briefly, the male and female fishes of length 3.25 ± 0.08 cm and weighing 1.15 ± 0.12 g were treated with different doses of CMEO (0–250 µg/mL) over 48 h and observed for any kind of toxicity symptoms inlcuding body color change, difficulty in swimming, and mortality [36].

### 2.10. Data Analysis

The percent mortality in different tests was analysed using a one-way ANOVA test at 5% significance with Grapes version 1.1.0 software [37]. Using Polo Plus 2.0 software, the LC_50_, LC_90_, slope and 95% confidence levels were calculated for both contact and fumigant toxicity according to Finney’s analysis. In the repellency test, to correct for heterogeneity of treatment variance, mean PR values were first transformed with an arcsine function before being assessed with one-way analysis of varience, and Tukey’s HSD analysis was used to separate means. The phytotoxicity study was represented as mean ± SD of germinating wheat grain, and tested by one-way ANOVA followed by Tukey’s HSD test.

## 3. Results

### 3.1. Chemical Configuration of CMEO by GC-MS

**Figure 1:**
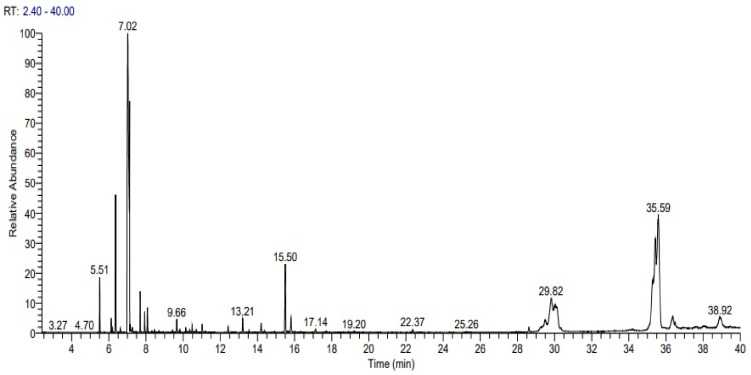
The GC-MS chromatograms of *Citrus maxima* essential oil (CMEO).

CMEO obtained through hydrodistillation had a yield of 0.58% ± 0.016 (*v*/*w*). Retention times (RTs) of each compound are listed. In total, we identified 25 different chemical compounds (Figure 1) from the oil (Table 1). The predominant components in *C. maxima* oil were D-limonene (33.61%), β-sitosterol (17.99%), α-sitosterol (12.19%), stigmasterol (5.22%) and α-pinene (4.32%).

### 3.2. Contact Toxicity

The results of contact toxicity bioassay indicated that CMEO at various concentrations had substantial contact toxicity via the residual film method to both *T. castaneum* and *C. maculatus* adult insects. CMEO caused more toxicity to adults of *C. maculatus* at 24 and 48 h after exposure, even at low concentrations (2–10 mg/cm^2^) when compared to *T. castaneum* (40–120 mg/cm^2^) (Table S1). The LC_50_ and LC_90_ values for CMEO against *T. castaneum* adults were 63.31 and 121.44 mg/cm^2^, respectively, after 24 h exposure (Table 2). Similarly, at 48 h exposure, LC_50_ and LC_90_ values for CMEO against *T. castaneum* adults were 37.15 and 109.38 mg/cm^2^, respectively. LC_50_ and LC_90_ values for *C. maculatus* adults were 7.12 and 16.73 mg/cm^2^, respectively, at 24 h. Similarly, at 48 h exposure, LC_50_ and LC_90_ values for *C. maculatus* adults were 5.06 and 13.44 mg/cm^2^, respectively. Increasing exposure times and concentrations resulted in a rise in mean mortality (%) with both stored grain insect pests. CMEO exhibited the highest efficacy against *C. maculatus* adults at lower concentration, followed by *T. castaneum* adults (Table 2).

### 3.3. Fumigant Toxicity

The CMEO exhibited fumigant potential against both stored grain insect pests. At various concentrations (1–10 mg/L air) for 24 and 48 h (Table S2). The CMEO showed high fumigant activity against *C. maculatus* adults compared to *T. castanaeum* adults. After fumigant toxicity bioassay, the LC_50_ and LC_90_ of the CMEO was calculated along with their respective confidence intervals (95%) (Table 3). The LC_50_ and LC_90_ for each exposure period were determined. Based on probit analysis, lethal concentrations of 4.95 mg/L and 4.13 mg/L resulted in 50% mortality (24 h and 48 h) in *T. castanaeum* adults. Similarly, lethal concentrations of 3.38 mg/L air and 1.34 mg/L air observed 50% mortality for *C. maculatus* adults (for 24 and 48 h), respectively. The effectiveness of CMEO was time-dependent. The results indicated that the lethal concentration decreased with increasing exposure periods. There was a greater vulnerability in *C. maculatus* adults compared with *T. castaneum* adults to CMEO (Table 3).

### 3.4. Repellent Activity

Both test insects, *T. castaneum* and *C. maculatus*, were strongly repelled by the CMEO (Table 4). According to the results of ANOVA, CMEO showed strong repellency to *T. castaneum* and *C. maculatus* adults (*p* > 0.05). At the lowest concentration (0.5 mg/cm^2^ dosage), mean PR values were over 50 % (Class III) at 2 h to 24 h post-exposure for *T. castaneum*. Notably, for *C. maculatus* adults, CMEO at the lowest concentration (0.5 mg/cm^2^) was able to cause repellence of only 23.90% (Class II) at 2 h to 24 h post-exposure. At higher concentration (5 mg/cm^2^), CMEO repelled *T. castaneum* with the mean PR of 78.7% (Class IV). The mean PR value was 69.30% (Class IV) at the highest applied concentration (5 mg/cm^2^) with *C. maculatus* adults. From the overall data, CMEO’s were moderately repellent (Class II–IV) to *C. maculatus* adults at 2 h to 24 h; whereas, the CMEO repelled *T. castaneum* adults, (Class III–V). The repellence activities of CMEO was significantly different against *T. castaneum* and *C. maculatus* (Table 4).

### 3.5. Larvicidal Potential of CMEO

The *Ar. Subalbatus*, *Ae. aegypti* and *Cx.* *tritaeniorhynchus* species of mosquito larvae were opted for larvicidal screening; the essential oil of *C. maxima* was least effective against the *Ar. subalbatus* species (76.24 ± 3.2 μg/mL). Conversely, the essential oil was effective against *Ae. Aegypti* and *Cx.* *tritaeniorhynchus* species (47.07 ± 2.4 and 58.04 ± 2.8 μg/mL) (Table 5).

### 3.6. Phytotoxicity Analysis of the CMEO

The phytotoxicity analysis of the essential oil was determined in terms of grain germination potentials. The Table 6 indicates no significant variation between any treated doses of CMEO on grain germination, and therefore was found to be non-phytotoxic.

### 3.7. Non-Targeted Organism Toxicity of CMEO

As indicated by the Table 7, no significant toxicity signs were observed in *P. reticulata* in terms of mortality, time spent on the top of water, or changes in body coloration. It is therefore confirmed that the CMEO has no toxic effects on the tested dose on non-targeted organisms.

## 4. Discussion

Many studies have reported that essential oils exhibit repellent and insecticidal effects against storage pests, and therefore may be considered an substitute method for managing stored grain insect pests [38,39]. In traditional medicine, essential oils were used to prevent pest infestations by extracting the oils from aromatic plants [40]. Due to their efficacy, economic value, and storage capabilities, these essential oils might be more effective than chemical pesticides for grain protection [41]. The FDA (Food and Drug Administration) recognizes the safety of botanicals over synthetic pesticides [13]. Since aromatic essential oils are highly volatile, they can control stored-product pests through contact, fumigant, and repellent actions [40,42]. In this respect, using essential oils offers an environmentally friendly and food-safe alternative to chemical insecticides.

The results indicated that essential oils of *C. maxima* peels were rich in D-limonene (33.61%), β-sitosterol (17.99%), α-sitosterol (12.19%), stigmasterol (5.22%) and α-pinene (4.32%). Furthermore, several reports showed that *C. maxima* peels are rich in D-limonene and α-pinene as their major chemical constituents [43,44,45]. The difference in oil yields and chemical composition could be explained by genotypic variations, environmental factors, and external factors such as climate, collection time, extraction methods and soil composition [46,47].

Essential oils derived from citrus wastes possess immense potential in the management of various insect pests and vectors; *C. limon* and *C. sinensis* were effective against *Aedes albopictus* [48], whereas, de Andrade Dutra, et al. [49] and Ribeiro, et al. [50] reported that *C. latifolia, C. reticulata*, and *Citrus sinensis* controlled the growth of *C. maculatus* and *Tetranychus urticae*. *C. limetta* was capable of killing the larvae of *Anopheles stephensi* [51], and *C. aurantifolia* essential oil was effective against *Aedes aegypti* [52]. Almost half of the orange material utilized for extraction of orange juice is discarded as waste [53]. Essential oils from citrus peels can be commercially formulated by utilizing nanotechnology [54]. Based on the essential oil yield from our study, one tonne of *C. maxima* peels can yield 5.8 litres of essential oil. The yield of CMEO reinforces its potential for exploitation as a commercial biopesticide. Essential oil yield obtained from *C. maxima* peels in the various studies ranges from 0.29–14.25% [55,56,57]. This variation can be due to the difference in extraction methods employed and genotypic variations, as well as abiotic factors.

Contact toxicity of analysis of *C. maxima* peels revealed a higher toxicitiy towards *C. maculatus* adults. Similar studies have also indicated the potential of *C. medica* essential oil as a contact toxic biopesticide against on *T. castaneum* adult insects [58]. Furthermore, some studies have shown that the essential oils of *Citrus* spp. had high contact toxicity against stored grain pests such as *C. maculatus* [49] and *Sitophilus zeamais* [59]. The CMEO revealed higher contact lethality to warehouse grain products than essential oils of *Pelargonium graveolens* [60], *Artemisia annua* [61] and *Melissa officinalis* [62]. In stored product insects, different constituents of essential oils may cause different toxic reactions due to the variance in the chemical configuration of CMEO and their weight and cuticle thickness. Limonene and α-pinene were major monoterpenes presented in *C. maxima* peels essential oil. Both these secondary metabolites are reported to have insecticidal [63], fumigant [64] and repellent properties from other citrus species [65]. For stored grain insects, cuticle damage caused by hydrocarbon absorption and abrasion can also result in death due to desiccation [66]. This study found that both *T. castaneum* and *C. maculatus* adults experienced toxicity from the bioactive molecules in the tested essential oils. This is because they may interact with target sites in the nervous system, respiratory system, and cutaneous system of insects [66].

The fumigant toxicity analysis indicated that CMEO showed significant mortality against pests of warehouse grains. However, CMEO’s were more toxic to *C. maculatus* adults than to *T. castaneum* adults. It has already reported that the essenitla oil of *C. sinensis* exerts higher sensitivity towards *C. maculatus* than *T. confusum* [67]. The results were not surprising as several studies have demonstrated *Citrus* spp. essential oil’s fumigant properties against *T. castaneum* [19]. *C. maculatus* [49,68], *Oryzaephilus surinamensis* and *S. zeamais* [69]. This result is in line with the fumigant effect of *Citrus reticulata* against *Cryptolestes ferrugineus* adults. Similarly, de Andrade Dutra, de Oliveira, Navarro and Santos [49] indicated the fumigant toxicity of different *Citrus* spp. against *C. maculatus* adults. We found that the LC_50_ value of *C. maxima* was 3.38 mg/L air against *C. maculatus* adults, which was better than that of the fumigant toxicity of essential oil from other *Citrus* spp.

Besides, the repellency of CMEO was tested against warehouse pests, and both test insects were repelled from essential oils from *C. maxima* peels. The essential oils extracted from *C. maxima* peels proved to be effective repellants against insect pests. Both *T. castaneum* and *C. maculatus* adults were repellent to the essential oils in a concentration-dependent manner. *Citrus* spp. have also been shown to repel storage insect pests such as the *T. castaneum* [58], *S. zeamais,* and *C. maculatus* [68]. Accordingly, the repellency of CMEO can be endorsed to the existence of D-limonene and α-pinene, which have been reported to be repellents [58,70]. The bioactivity of individual essential oil components of *C. maxima*, as well as their interactions with storage pests, should be further evaluated. In addition, the level of repellency has a direct effect on the reduction of oviposition, as well as the development of adults [71]. Hence, the essential oil of *C. maxima* can be used as a repellent biopesticide against pests of warehouse grains.

The majority of the bioactive chemicals found in essential oils had significant effects on non-target fishes, including fishes, or on human health or the environment. These essential oils have a higher efficiency and could serve as biopesticides in the future, as well as serving as better tools for integrated pest management because of their eco-friendliness [72].

## 5. Conclusions

The study concludes that the peel essential oil extracted from the *Citrus maxima* is highly useful as a biologically safe pesticide against the common stored grain insect pests *T. castaneum* and *C. maculatus*. Further, potent larvicidal toxicity against different mosquitoe species was observed, which may increase the economic value of the essential oil. Considering also the non-toxic nature on germinating grains and non-targeted organisms like fishes extends the possible application of the essential oil as a biologically safe green pesticide, even in the storage of food and seed grains.

## Figures and Tables

**Table 1:** Chemical configuration of *Citrus maxima* essential oil (CMEO).

Peak No.	Retention Time	Component	RSI ^a^	%RA ^b^
1	5.51	3-carene	934	1.49
2	6.12	Cyclohexene, 4-methylene-1-(1-methylethyl)	921	0.40
3	6.36	α-Pinene	895	4.32
4	7.02	D-Limonene	881	33.61
5	7.69	trans-Linalool oxide (furanoid)	878	1.17
6	7.93	cis-Linaloloxide	901	0.62
7	8.08	1,6-Octadien-3-ol, 3,7-dimethyl	921	0.76
8	9.66	α-Terpineol	923	0.57
9	10.48	2,6-Octadienal, 3,7-dimethyl-, (Z)	876	0.31
10	11.02	Citral	921	0.37
11	12.42	2-Carene	883	0.26
12	13.21	Geranyl acetate	917	0.64
13	14.20	Caryophyllene	921	0.41
14	15.49	α-Cubebene	882	3.18
15	15.81	α-Guaiene	904	0.90
16	29.50	4,4-dimethyl Cholesta-22,24-dien-5-ol	895	0.82
17	29.82	Stigmasterol	917	5.22
18	30.05	Desmosterol	864	3.79
19	30.12	(3á,22E) 3-methoxy-Stigmasta-5,22-diene,	873	1.95
20	35.29	campesterol	895	4.31
21	35.43	α-Sitosterol	838	12.19
22	35.59	β-Sitosterol	928	17.99
23	36.36	24-propylidene-, (3á) Cholest-5-en-3-ol,	874	1.90
24	36.49	Allopregnane-3á,7à,11à-triol-20-one	829	0.39
25	38.91	9,19-Cyclolanost-24-en-3-ol, (3á)	898	2.41
Total (%)				100.00

^a^ Reverse similarity index; ^b^ Relative area.

**Table 2:** Lethal concentrations on contact activity of CMEO against various insect pests.

Test Insects	Exposure (h)	LC_50_ ^a^ (mg/cm^2^)	LC_90_ ^a^ (mg/cm^2^)	Slope ± SEM ^b^	χ^2^ (d.f)
*T. castaneum*	24	63.31 (49.4–73.2)	121.44 (106.2–151.5)	0.022 ± 0.004	0.335 (3)
	48	37.15 (18.8–48.2)	109.38 (86.6–192.8)	2.733 ± 0.708	0.264 (3)
*C. maculatus*	24	7.12 (5.1–12.7)	16.73 (10.4–153.2)	3.458 ± 0.601	4.58 (3)
	48	5.06 (3.3–7.1)	13.44 (8.8–52.2)	3.023 ± 0.501	3.64 (3)

^a^ Values in parenthesis represent lower and upper confidence limit; ^b^ SEM: Standard error of mean; χ^2^: Chi-square.

**Table 3:** Lethality of CMEO by fumigation against *T. castaneum* and *C. maculatus*.

Test Insects	Exposure Time (h)	LC_50_ (mg/L Air)	LC_90_ (mg/L Air)	Slope ± SEM ^a^	χ^2^ (d.f)
*T. castaneum*	24	4.95 (4.1–5.9)	12.68 (9.7–20.1)	3.138 ± 0.503	1.396 (3)
	48	4.13 (3.3–5.0)	11.82 (8.9–18.8)	2.806 ± 0.431	1.262 (3)
*C. maculatus*	24	3.38 (2.1–4.8)	29.61 (14.8–170.5)	1.360 ± 0.318	1.136 (3)
	48	1.34 (0.35–2.2)	17.80 (9.1–129.6)	1.144 ± 0.316	2.535 (3)

^a^ SEM: Standard error of mean; χ^2^: Chi-square.

**Table 4:** Repellecy of CMEO against pests of stored grains at different exposure times.

Test Insects	Concentration (mg/cm^2^)	Repellence Percentage of Treatments After					% Repellency (Mean± ^b^SEM)	Repellent Class
		2 h	4 h	6 h	12 h	24 h		
*T. castaneum*	0.5	33.3 ± 13.3 ^a^	46.7 ± 6.6 ^a^	60.0 ± 11.5 ^a^	66.7 ± 6.6 ^a^	60.0 ± 11.5 ^a^	53.3 ± 5.8 ^b^	III
	1.5	33.3 ± 17.6 ^a^	66.7 ± 6.6 ^a^	60.0 ± 11.5 ^a^	66.6 ± 6.6 ^a^	66.7 ± 13.3 ^a^	58.7 ± 6.5 ^ab^	III
	2.5	60.0 ± 11.5 ^a^	53.3 ± 6.6 ^a^	66.7 ± 13.3 ^a^	73.3 ± 6.6 ^a^	73.3 ± 17.6 ^a^	65.3 ± 3.8 ^ab^	IV
	3.5	66.7 ± 13.3 ^a^	60.0 ± 11.5 ^a^	73.3 ± 17.6 ^a^	80.0 ± 11.5 ^a^	86.7 ± 13.3 ^a^	73.3 ± 4.7 ^ab^	IV
	5	73.3 ± 6.6 ^a^	66.7 ± 13.3 ^a^	73.3 ± 13.3 ^a^	86.7 ± 13.3 ^a^	93.3 ± 6.6 ^a^	78.7 ± 4.9 ^a^	IV
	F value	2.07	0.85	0.29	1.02	1.29	3.86	
	*p* value	0.16	0.53	0.87	0.44	0.33	0.02	
	^a^ d.f	4	4	4	4	4	20	
*C. maculatus*	0.5	6.7 ± 6.6 ^a^	13.3 ± 6.6 ^a^	33.3 ± 17.6 ^a^	33.3 ± 13.3 ^a^	33.3 ± 6.6 ^b^	23.9 ± 8.9 ^c^	II
	1.5	20.0 ± 11.5 ^a^	26.7 ± 6.6 ^a^	40.0 ± 11.5 ^a^	53.3 ± 6.6 ^a^	53.3 ± 17.6 ^ab^	38.7 ± 6.7 ^bc^	II
	2.5	40.0 ± 11.5 ^a^	33.3 ± 13.3 ^a^	53.3 ± 17.6 ^a^	60.0 ± 11.5 ^a^	60.0 ± 11.5 ^ab^	49.3 ± 5.4 ^abc^	III
	3.5	46.7 ± 6.6 ^a^	53.3 ± 13.3 ^a^	60.0 ± 11.5 ^a^	80.0 ± 11.5 ^a^	86.7 ± 6.6 ^a^	65.3 ± 7.7 ^ab^	IV
	5	40.0 ± 11.5 ^a^	60.0 ± 19.9 ^a^	80.0 ± 11.5 ^a^	73.3 ± 13.3 ^a^	93.3 ± 6.6 ^a^	69.3 ± 9.1^a^	IV
	F value	3.13	2.30	1.69	2.50	5.18	7.01	
	*p* value	0.06	0.12	0.22	0.10	0.01	0.001	
	^a^d.f	4	4	4	4	4	20	

^a–c^ Means having the same superscript show no significant variation (*p* < 0.05). ^a^d.f: Degree of freedom, ^b^SEM: Standard error of mean.

**Table 5:** Efficacy of the essential oil from *C. maxima* peels as a potential mosquito larvicidal agent (LC_50_ value expressed in μg/mL).

Mosquito	LC_50_ (μg/mL)	Slope ± SEM	χ^2^ (d.f)
*Ar. subalbatus*	76.24 ± 3.2	0.6566 ± 0.027	1.439 (3)
*Ae. aegypti*	47.07 ± 2.4	1.0643 ± 0.052	5.855 (3)
*Cx. tritaeniorhynchus*	58.04 ± 2.8	0.8630 ± 0.042	2.099 (3)

**Table 6:** Effect of CMEO on the ability of wheat germination (% germination).

Duration (Hours)	% Germination in Untreated Grains	*C. maxima* Essential Oil (µg/mL)		
		50	100	250
48	12.4 ± 1.0	13.1 ± 0.7 ^n^	12.2 ± 0.9 ^n^	11.9 ± 1.2 ^n^
72	30.7 ± 0.9	31.3 ± 0.6 ^n^	30.8 ± 1.2 ^n^	31.4 ± 1.2 ^n^
96	58.5 ± 1.5	59.2± 1.4 ^n^	59.9± 1.1 ^n^	58.4 ± 2.1 ^n^
120	80.6 ± 1.7	82.1 ± 1.6 ^n^	81.4 ± 1.4 ^n^	80.9 ± 2.1 ^n^
144	93.4 ± 2.1	92.5 ± 1.6 ^n^	93.1 ± 1.5 ^n^	92.4 ± 2.5 ^n^

(^n^ indicate no significant variation with untreated grains at *p* < 0.05).

**Table 7:** Effect of CMEO on the non-targeted organism (*P. reticulata)* and changes in the signs and symptoms of toxicity, as well as the mortality rate.

CMEO Dose (µg/mL)	% Mortality	Number of Fish Having Swimming Difficulty	Fishes Having Any Changes in Color	Total Time Spent on Top of Water (Seconds)
0	0	0	0	27.6 ± 3.0
50	0	0	0	27.4 ± 3.0
100	0	0	0	29.7 ± 2.0
200	0	0	0	26.8 ± 3.0
250	0	0	0	29.5 ± 3.0

## Data Availability

Data may be made available on valid request.

## Supplementary Materials

The following supporting information can be downloaded at: https://www.mdpi.com/article/10.3390/insects13050480/s1. Table S1: Efficacy of contact toxicity of CMEO against *T. castaneum* and *C. maculatus* at different exposure times; Table S2: Efficacy of fumigant toxicity of CMEO against *T. castaneum* and *C. maculatus*.
