# Supplementary material for: Utilization of Pomelo (Citrus maxima) Peel Waste into Bioactive Essential Oils: Chemical Composition and Insecticidal Properties
Source: Insects. 2022 May 20;13(5):480. doi: 10.3390/insects13050480 (PMC9146202; doi:10.3390/insects13050480)
Supplement: Supplementary file 1 [file insects-13-00480-s001.zip › insects-1650673-supplementary.pdf]

Contact toxicity

Supplementary Table S1. Efficacy of contact toxicity of CMEO against *T. castaneum* and *C. maculatus* at different exposure times

**Test insects**	**Dose (mg/cm^2)**	**Mean mortality per cent of treatments after**	
		**24 h**	**48 h**
T. castaneum	40	36.7±3.3b	76.7±6.6bc
	60	46.7±8.8b	83.3±3.3ab
	80	50.0±11.5b	70.0±5.7c
	100	80.0±5.7a	83.3±3.3ab
	120	90.0±5.7a	93.3±3.3a
	Control	0±0c	0±0d
	F value	21.54	62.80
	P value	0.00	0.00
C. maculatus	2	6.7±3.3de	16.7±3.3cd
	4	13.3±3.3d	23.3±3.3c
	6	36.7±3.3c	50.0±10.0b
	8	50.0±5.7b	70.0±10.0ab
	10	83.3±3.3a	90.0±5.7a
	Control	0±0e	0±0d
	F value	77.13	24.30
	P value	0.00	0.00

*Means within the same raws followed by same letter are not significantly different ($p<0.05$)

Fumigant toxicity

Supplementary Table S2. Efficacy of fumigant toxicity of CMEO against *T. castaneum* and *C. maculatus* at different exposure times

**Test insects**	**Dose (mg/L air)**	**Mean mortality per cent of treatments after**	
		**24 h**	**48 h**
T. castaneum	1	3.3±6.7d	6.7±3.3d
	3	20.0±13.3cd	23.3±3.3c
	5	33.3±5.5bc	53.3±6.6b
	7	50.0±6.6b	60.0±0b
	10	86.7±3.3a	93.3±3.3a
	Control	0±0d	0±0d
	F value	32.07	151.31
	P value	0.00	0.00
C. maculatus	1	30.0±0c	53.3±8.8b
	3	43.3±6.6bc	56.7±8.8b
	5	56.7±6.6ab	70.0±5.7b
	7	63.3±3.3ab	73.3±6.6a
	10	76.7±6.6a	96.7±3.3c
	Control	0±0d	0±0d
	F value	40.26	25.70
	P value	0.00	0.00

*Means within the same raws followed by same letter are not significantly different ($p<0.05$)
